# Reframing creativity in the city: on the emergence of contemporary township creativity

**DOI:** 10.1080/00343404.2025.2495797

**Published:** 2025-05-22

**Authors:** Irma Booyens

**Affiliations:** a Department of Work, Employment and Organisation, Strathclyde Business School, University of Strathclyde, Glasgow, UK; b School of Tourism and Hospitality, University of Johannesburg, Johannesburg, South Africa

**Keywords:** urban informality, creative cities, ordinary neighbourhoods, townships, creative entrepreneurs, cultural production, D0, D20, E20, F60

## Abstract

This paper offers a provocation for reframing the spatiality of urban creativity by interrogating the groundswell of contemporary creativity in South African townships. Creativity in marginalised neighbourhoods such as townships does not fit dominant creative city narratives and global city aspirations. The literature is largely silent about creative work and spaces characterised by informality. This paper draws on critical urbanism emphasising the ‘ordinary’ and ‘informal’ to reinforce urban theory based on the neglected realities of Global South cities. The roles of tourism and actors in township creativity are examined and associated risks, vulnerabilities and potentialities are also considered.

## 1. INTRODUCTION

The creative city literature remains mainly informed by Global North experiences (Mbaye & Dinardi, 2019; Scott, 2014). Although limited urban creative economy literature is forthcoming from the Global South, there is a particular dearth of research vis-à-vis African contexts (Mbaye & Pratt, 2020). There is a growing recognition that creative cities are heterogeneous and that creative urbanisation processes play out differently in different contexts. A ‘second wave’ of creative city research, adopting postcolonial and relational approaches to urban analysis, emphasises the ‘situated experiences’ and ‘varied local effects’ of creative city-making in the Global South (Acuto et al., 2019; Mbaye & Pratt, 2020; Nkula-Wenz, 2019; Sitas, 2020). Research on lived urbanism emphasises the value of culture in contemporary cities to create jobs and a sense of meaning, worth and identity in communities while highlighting the social nature of the creative economy and the work of communities of practice, civil society organisations and creative intermediaries (Comunian & England, 2022; Mbaye & Pratt, 2020; Sitas, 2020; Thiel, 2017).

South Africa’s main cities, particularly Johannesburg and Cape Town, have adopted global city aspirations and creative city strategies (Booyens, 2012; Minty & Nkula-Wenz, 2019; Nkula-Wenz, 2019; Rogerson, 2006). Innovation and specialisation are shown to drive creative clustering in large, global cities (Adler & Florida, 2021; Florida, 2002). However, global city perspectives tend to be spatially blind, thus failing to consider the peculiarities of Global South cities (Nuccio & Pedrini, 2014). Moreover, global city centrality is increasingly questioned by ‘disruptive exceptions’ challenging normative assumptions (Peck, 2015). Township creativity presents such an exception. As a result of segregation by design, townships were earmarked for people of colour and located ‘in outer areas or in-between spaces’ of cities (Lynge et al., 2022, p. 621). Despite service delivery improvements since the end of apartheid, townships remain characterised by structural unemployment, relative deprivation and many social ills (Charman et al., 2020; Rogerson, 2019; Todes & Turok, 2018). In recent years, a groundswell of creativity has occurred in the townships of Soweto (Johannesburg) and Langa (Cape Town) involving (mostly) young creative entrepreneurs making creative products and offering creative experiences (Booyens & Rogerson, 2015, 2019a, 2019b; Charman, 2016; Gregory & Rogerson, 2018). These townships also boast creative spaces and a variety of cultural events (Booyens & Rogerson, 2019a, 2019b). Despite recognising that the spatiality of creativity is not confined to central cities but is also found in suburban and non-metropolitan areas (Chapain et al., 2013; Collis et al., 2013; Scott, 2014), the literature is largely silent about creativity by informal creative workers in impoverished neighbourhoods such as townships, marginal to creative urban economies.

Research on peripheral urbanisation emphasises the differing modes of production between cities in the Global North and South. Importantly, peripherality ‘does not simply refer to a spatial location in the city – its margins – but rather to a way of producing space that can be anywhere’ (Caldeira, 2017, p. 4). Informality, the norm in the Global South, produces ‘everyday’ urban practices and places (Pratt, 2019; Roy, 2009). Critical urbanism stresses the importance of the ‘ordinary’ (or ‘everyday’) to bolster urban theory based on the neglected realities of cities (Parnell & Oldfield, 2014; Peck, 2015). However, the notion of informality is an uneasy fit with urban theory. Informality is associated with uneven geographies of spatial value and typically results in ‘othering’ (Acuto et al., 2019; Roy, 2009). At the same time, however, an emphasis on informality reveals ‘hidden’ urban realities and experiences (Parnell & Oldfield, 2014). Importantly, Pratt (2019, p. 614) urges ‘to really “see” informality, we must resist generalisation: there are only particular timed and placed informalities: Each informality exists in relation to a wider (local) social, political and economic setting, as well as global one’.

There is a need to go beyond ‘disciplinary boundaries that limit informal urbanism to the study of housing or the labour market’ (Acuto et al., 2019, p. 475). Indeed, oversimplifications of the ‘ordinary’ make invisible other forms of informality (Cirolia & Scheba, 2019). This paper foregrounds township creativity as an overlooked feature of urban informality. Accordingly, it positions township creatives as ‘ordinary’ residents and (mostly) informal workers who eke out their livelihoods from their creative endeavours in ‘ordinary’ neighbourhoods, that is, the townships of Soweto and Langa. This paper explores why contemporary creativity emerged in townships. It builds on some 15 years of township tourism and urban creativity research in Johannesburg and Cape Town and further interprets the findings of three research projects (see the Methods below) to inform the analysis presented in this paper. It employs an evolutionary lens to interrogate (1) the role of tourism in the rise of contemporary forms of township creativity; and (2) the roles of local actors – individuals and organisations – in shaping cultural production, creative spaces and area change in townships. As per evolutionary economic geography theory, economic activities are contextual, dynamic and dependent on the interplay between evolutionary-formed structures coupled with individual behaviour (Boschma & Frenken, 2006). Furthermore, the paper discusses critical considerations concerning township creativity, including the associated risk of creative-led gentrification, the precarity of township creatives and the liminal potentialities of township spaces.

## 2. LITERATURE

### 2.1. Global cities and creativity in South Africa’s main cities

The growth of creative industries is characteristic of service sector expansion in post-industrial economies (Baum, 2020; Richards, 2011). Mirroring global city patterns, a structural economic change has occurred in the economic landscape of South Africa’s main cities with urban economies shifting from mining and manufacturing to services (Bhorat et al., 2018). Accordingly, services-led growth has become a main feature in the country’s largest cities – the tertiary sector contributes up to 75% and 80% to the economies of Johannesburg and Cape Town, respectively (Cheruiyot, 2018; City of Cape Town, 2016). After financial services, tourism and creative industries constitute a distinct geographical trajectory in both cities (Cheruiyot, 2018).

In the literature, global cities and creative cities are often used as interchangeable terms (Baum, 2020). Global city ‘making’ (or ‘worlding’) is the aim of creative city strategies pushing local authorities to adopt neoliberal policies in the form of entrepreneurial governance and market-led development (Nkula-Wenz, 2019; Roy, 2009). In turn, creative city branding harnesses the power of the arts to ‘attract new development, generate consumption (and sales tax), and boost real estate values’ (Grodach, 2016, p. 23). In South Africa, creative city strategies largely correspond with neoliberal growth tendencies and global city aspirations, mimicking urban development approaches in the Global North (Booyens, 2012; Minty & Nkula-Wenz, 2019); however, there is a disconnect when it comes to townships (as stressed in this paper). Creative economy policies have been adopted at the national level in South Africa, and the potential of culture and creativity as drivers of local development is firmly on the radar of provincial and municipal governments alike, particularly in the country’s largest metros (metropolitan municipalities) of Johannesburg and Cape Town (Nkula-Wenz, 2019, p. 586).

City-based creativity shares a close relationship with tourism through place-based production and consumption. Tourism can be regarded as a cultural product of globalisation (Baum, 2020). ‘Symbolic’ economies involve the consumption of goods and services, and meanings and cultural forms (Crewe & Beaverstock, 1998). Creative-led branding strategies to give cities a ‘symbolic edge’, typically rely on ‘soft’ location factors such as culture, lifestyle, leisure and tourism as distinguishing features (Richards, 2011; Scott, 2014). Therefore, the rise of creativity in global cities is linked to the growth of the experience economy driven by tourism through the ‘development of specific experience environments and the repackaging of a range of tourist services as experiences’ drawing on arts and culture (Richards, 2011, p. 1223). Tourist consumption of creativity takes on a spatial dimension in city spaces where creative sectors cluster. Visitors flock to creative urban areas because of the ‘bohemian’ atmosphere and ‘cool’ image, and tourist consumption of creative places ensues when trendy cafés and restaurants, boutique hotels and artistic shops surface (Pappalepore et al., 2014; Pratt, 2009).

As per classic post-Fordist creative city narratives, creative firms characteristically cluster in declining inner-city working-class neighbourhoods where manufacturing has collapsed (Grodach, 2016; Pratt, 2009; Scott, 2014). Skilled (upper- and middle-class) creative professionals are drawn to these areas because of their affordability and proximity to city centres to establish creative firms in redeveloped industrial premises (former derelict factories and warehouses) (Florida, 2002; Pratt, 2009; Scott, 2014). Creative city strategies promoting private investment in creative urban redevelopment are criticised for driving gentrification and, accordingly, the exclusion of low-income families from deprived neighbourhoods (Grodach, 2016; Nuccio & Pedrini, 2014; Scott, 2014). Because arts-based urban change is highly visible, gentrification is an assumed inevitable outcome (Grodach, 2016; Markusen, 2006). However, Grodach (2016) contends that the arts have a divergent and conflicting relationship with place depending on the context. While the problematic consequences of creative urban regeneration have been studied widely, the outcomes in low-income neighbourhoods (such as townships) have received less attention (Grodach, 2016; Nuccio & Pedrini, 2014). Critiques concerning exclusion associated with creative-led regeneration tend to regard culture ‘merely as a vehicle for social and urban space change’ (Thiel, 2017, p. 23). Such views typically focus on creative individuals and urban environments while ignoring the complexity of cultural production and its role in labour markets, infrastructures, organisations and institutions (Thiel, 2017). Roy (2009) argues that when the production of space is understood through the lenses of urban development and gentrification, the symbolic economies of the cities, important for new geographies of urban theory, are often neglected.

### 2.2. Peripheral urbanisation, heterotopia and cultural production in marginalised spaces

Peripheral urbanisation consists of interrelated processes emphasising the agency of residents, the temporality of processes, the unsettling of official logics and the generation of new forms of politics to create often highly unequal and heterogeneous cities (Caldeira, 2017). Follmann et al. (2023) stress that urbanisation processes in the Global South are dynamic and diverging, consisting of multiple dimensions of *connectivity* and *bypass* and processes of *fragmentation* and *integration*.

In the Global South, informality is regarded as a mode in the production of urban space, unpinned by negotiations and temporality concerning the constant transformation of both politics and spaces (Follmann et al., 2023; Porreca & Janoschka, 2024; Pratt, 2019). Informality does not necessarily pertain only to unregulated economic activities or informal settlements; instead, it is structured through various forms (Roy, 2009). Roy (2009, p. 826) stresses that ‘The differential value attached to what is ‘formal’ and what is ‘informal’ creates the patchwork of valorized and devalorized spaces’, which in turn produces (or reinforces) uneven urban spatial development. *Fragmentation* is widely used to highlight the challenges of uneven development – both socio-spatial and institutional fragmentation produce regulatory vacuums and ambiguities (Follmann et al., 2023). The *bypass* mode of urbanisation, a divergence from integration and urban connectivity, produces extremely uneven development and socio-spatial segregation (Follmann et al., 2023). While urban studies regularly centre on extreme inequalities between the destitute and affluent, most neighbourhoods are simply ‘in-between’ (Sitas, 2020) or just ‘ordinary’ in other words. Despite long-term deprivation in certain neighbourhoods, gradual economic and physical upgrading, also evident in the townships of Soweto and Langa (see the Context section), leads to the slow ‘encroachment of the ordinary’ (Bayat, 2000, p. 533).

Avni and Yiftachel (2014, p. 488) suggest that the gap between formal and informal is ‘increasingly blurred and pervasive’ in marginalised urban spaces. According to Foucault (1986/2008), ‘other’ spaces or ‘heterotopia’ are made up of juxtaposed single places with multiple, often incompatible, social spaces. Heterotopia exposes ‘real’ places and envisions public life as temporal rather than fixed. With an emphasis on place, heterotopias can be understood as liminal realms containing multiple binary and non-binary states (Banfield, 2022). While liminal realms are uncomfortable, ambiguous and transitory spaces characterised by chaos and uncertainty, they are also transformative spaces of possibility (Banfield, 2022; Follmann et al., 2023; Lancione & Simone, 2021). However, residents who are ‘far from powerless’ use such liminal spaces as bases for self-organisation and empowerment (Avni & Yiftachel, 2014, p. 489). Indeed, personal resourcefulness characterises the developmental possibilities of third-wave cities (Scott, 2014). While power relations remain skewed in favour of the political elite and the wealthy, the agency of ordinary residents (often invisible) is significant for theory and policy since their actions contribute to the shaping of city spaces (Avni & Yiftachel, 2014).

As with the concept of informality, liminality in urban studies is usually applied to housing and political activism (Lancione & Simone, 2021). Liminality vis-à-vis cultural production in marginalised spaces, therefore, is underexamined. Moreover, there is limited literature on creativity in impoverished Global South neighbourhoods. This said a few notable examples include creative *kampung*s (informal settlements) in Tangerang City (Indonesia) (Lestari et al., 2023), and creativity in impoverished urban areas in Dakar (Senegal) and Rio de Janeiro (Brazil) (Mbaye & Dinardi, 2019; Müller, 2017), and South African townships (Booyens, 2021; Booyens & Rogerson, 2019a, 2019b; Gregory & Rogerson, 2018; Montanini, 2020). These neighbourhoods feature street art, cultural festivals, community arts, including music and theatre groups, and creative visitor experiences (Booyens & Rogerson, 2019a; Mbaye & Dinardi, 2019; Müller, 2017). These examples of creativity also include forms of cultural production. At the heart of cultural production are the ‘reflexive subjects’, that is, actors operating a distance away from the standard rules and resources who observe their environments and adapt to the circumstances to which they are exposed (Lash & Urry, 1994; Thiel, 2017). An emphasis on cultural production further highlights the activities of organisations supporting creative workers (Comunian, 2011), that is, local governments funding cultural spaces in struggling neighbourhoods (Grodach, 2016; Minty & Nkula-Wenz, 2019; Rauws, 2016) and non-governmental organisations (NGOs) supporting community arts/grassroots initiatives (Mbaye & Dinardi, 2019; Sitas, 2020). NGOs often take on the role of cultural intermediaries, acting as brokers between creatives and consumers while also facilitating cultural development and providing entrepreneurial support (Comunian & England, 2022; Hracs, 2015).

Baum (2020) draws attention to low-skilled workers in the creative economy, emphasising that labour market changes in cities result in an overlap between low-skilled service and creative work. This phenomenon does not fit the creative city policy – and ‘creative class’ – rhetoric (Florida, 2002) concerning highly skilled and professional creative workers (Baum, 2020; Markusen, 2006; Scott, 2014). ‘Ordinary’ workers doing informal, low-skilled service and creative work are largely ‘invisible’ and ‘anonymous’ in cities (Baum, 2020) since they tend to operate outside of creative districts, owing to their marginality and the liminality of their work. Research on informal (creative) entrepreneurs underscores the practice of ‘hustling’ to navigate precarity, access finance and operate in peripheral markets (Cooper, 2021, 2025; Steedman & Brydges, 2023). This practice highlights the agency of creatives as individual actors who not only shape their livelihoods, but also engage relationally with cultural production and place (Kourtit & Nijkamp, 2019; Lash & Urry, 1994; Thiel, 2017).

### 2.3. An evolutionary perspective on area development

Evolutionary perspectives highlight the complexity of economic systems. Economic development, accordingly, is neither monolithic and linear nor tied to a particular point in time, but instead, an ongoing and often unpredictable process. Furthermore, multi-actor and multidimensional processes shape economic development on a spatial level. Boschma (2017, p. 354) explains that new economic activities (or novelty) ‘draw on and combine local capabilities’. Local capabilities include core assets, competencies or resources characteristic of an area or region (Malecki, 2010). Relatedness – new activities drawing on and combining local capabilities – accordingly drive economic diversification in areas and regions (Boschma, 2017). In the context of the creative economy, cultural heritage assets (cultural amenities and historical monuments) are significant contemporary local capabilities for stimulating new urban initiatives (Comunian, 2011; Kourtit & Nijkamp, 2019). In addition, Kourtit and Nijkamp (2019) point to the synergies between urban amenities with a high cultural heritage value and their power to attract people (visitors) and firms to places.

The emergence of economic novelty and its impact on area development and change is path dependent, that is, historical factors pave the way for economic development trajectories often leading to complementary or contesting paths (Martin, 2009). Path creation is a mechanism for ‘de-locking’ to create new development paths. While new paths may result from chance events or historical ‘accidents’, path creation is further influenced by actors (including institutions) who make strategic decisions to bring about change and implement economic novelty (Boschma & Martin, 2007; MacKinnon et al., 2022; Martin & Sunley, 2007).

The behaviours of individual actors are typically unplanned and unpredictable, yet individual adaptation often results in self-organising spatial structures and acts as a stimulus for broader development (Boschma & Martin, 2007; Martin & Sunley, 2007). In correspondence, Russell and Faulkner (1999) emphasise the collective impact of a few individuals (i.e., movers and shakers or chaos makers) on (tourism) area development. A cumulative, non-linear outcome of individual adaptation, therefore, is the creation of new order (and institutions) on a spatial level (Meekes et al., 2017a; Rauws, 2016). However, Meekes et al. (2017a) maintain that while bottom-up processes shape the leisure sector, development is further aided by governmental and non-governmental (formal and informal) organisations.

## 3. CONTEXT

Soweto and Langa are long-established settlements, earmarked specifically for Black African residents under colonial rule and apartheid. Orlando was established as a ‘Native location’ on the south-western periphery of Johannesburg in 1932 (Magubane & Lee, 1979). In 1954, the apartheid government forcefully removed Black residents from the city’s central suburbs, as part of its ‘slum’ clearance programme, to Meadowlands and Diepkloof, north-west and east of Orlando (Magubane & Lee, 1979). These areas were collectively named Soweto (Southwestern Townships) in 1963. Similarly, Langa was established in 1927 on the site of the former Ndabeni ‘location’ (*c.*1901) (Booyens et al., 2021).

As part of post-apartheid reforms, urban townships became part of the greater metropolitan (metro) municipality areas of large cities such as Johannesburg and Cape Town (Pillay, 2004). Established townships such as Soweto and Langa have benefitted from upgrading and improved basic service delivery since the reforms of the 1990s promoting ‘redress and redistribution by favouring development in marginalised areas’ (Todes & Turok, 2018, p. 9). This is illustrative of local governments responding to the directives of the post-apartheid state and adopting a welfare logic to redress the challenges of social exclusion and improve basic service delivery, including the provision of social housing, especially in townships (Parnell & Robinson, 2012). Average incomes Langa and Soweto range from low to lower-middle incomes – unemployment rates are high regardless – and there is a mix of housing structures, including brick-and-mortar houses and informal (or ‘shack’) dwellings (Lynge et al., 2022; Scheba & Turok, 2020). Urban sprawl over the last three decades has resulted in new townships emerging on outer urban peripheries (Lynge et al., 2022). These areas, inhabited by domestic and foreign migrants alike, are marked by extreme poverty and vulnerability and poor services overall.

Although townships such as Langa and Soweto are better located and serviced than new townships on the outer peripheries, fragmentation dynamics (Follmann et al., 2023) are evident in townships, albeit to varying degrees. The largely informal economies of townships remained disconnected from formal economies and city spaces because of the stark spatial divisions and inequalities in South African cities – a lingering legacy of apartheid rather than the impact of global urbanisation (Parnell & Robinson, 2012). Overall, townships lack robust industrial and formal business bases (Charman et al., 2020; Lynge et al., 2022; Rogerson, 2019). Economic activities in townships tend to be informal, small-scale and survivalist, with self-employment as a prevalent feature (Charman et al., 2020; Rogerson, 2019). Street trading and *shebeens* (taverns) are typical examples of township enterprises, along with services such as hairdressing, shoe repair and tailoring, and limited retail development and small manufacturing (welding, construction and furniture manufacturing) in some of the largest townships (Charman et al., 2020; Ngwenya & Zikhali, 2018; Rogerson, 2019; Todes & Turok, 2018). The shortage of formal businesses is a significant gap in the economic base of townships. Barriers constraining township businesses include limited access to basic services, financial resources, skills, infrastructure, public transport, markets and client bases, and high levels of crime (Charman et al., 2020; Ngwenya & Zikhali, 2018; Scheba & Turok, 2020). As a result, townships remain poorly connected to global markets, talents and financial flows despite the global-city aspirations of South Africa’s main cities. In recent years, the cities of Johannesburg and Cape Town, with the support of provincial governments, have been most active in developing strategies to boost economic development in townships, that is, building ‘inclusive’ township economies and developing townships as ‘viable urban locations’ (Rogerson, 2019; Scheba & Turok, 2020). Stimulating entrepreneurship and informal employment are key considerations for economic development in townships (Charman, 2016; Rogerson, 2019). Yet, informal creative township entrepreneurship has received limited, if any, policy or research attention.

**Figure 1.**
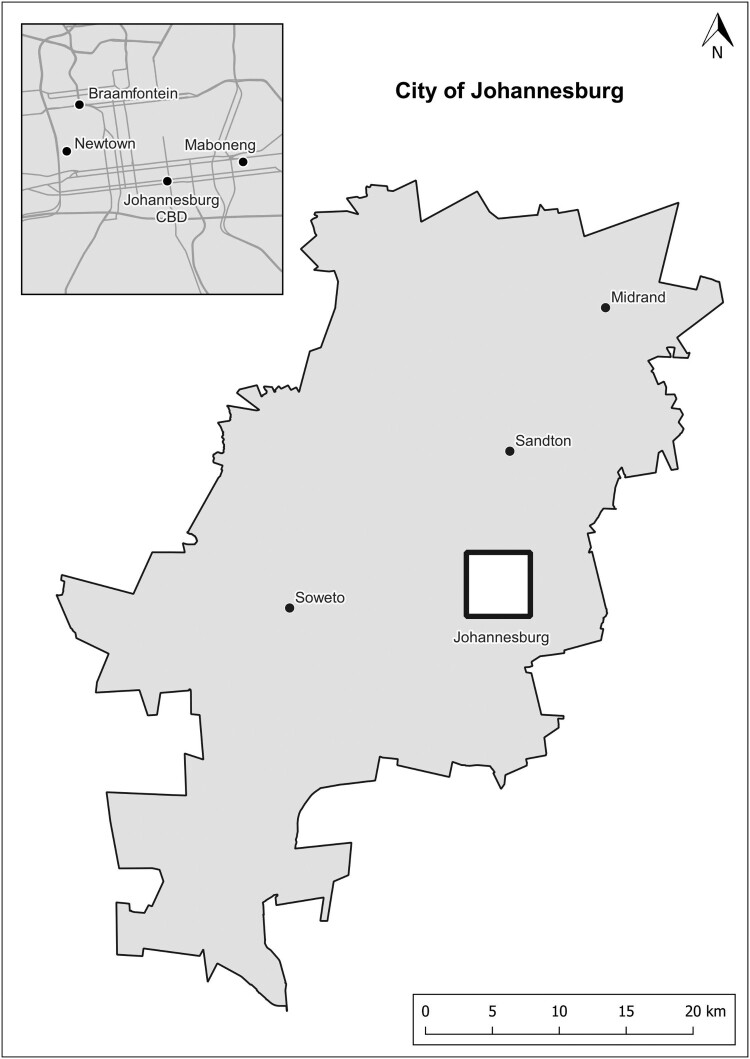
Locations of inner-city creative clusters and Soweto in Johannesburg, South Africa. Source: Author.

**Figure 2.**
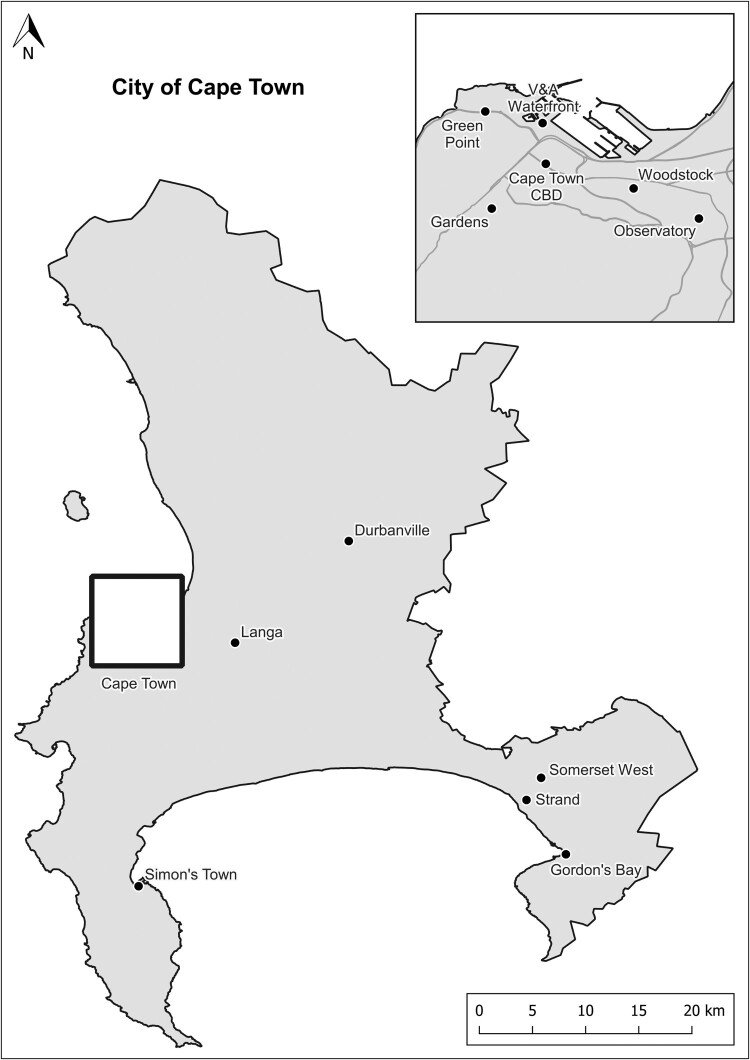
Locations of inner-city creative clusters and Langa in Cape Town, South Africa. Source: Author.

Townships such as Soweto and Langa have distinct local resources positioning these neighbourhoods firstly as urban cultural heritage destinations and secondly as creative neighbourhoods, as I will argue in this paper. Soweto is iconic for its political prominence in the struggle against apartheid and its vibrant urban culture in terms of arts, music and entertainment (Booyens, 2021; Booyens & Rogerson, 2019a; Magubane & Lee, 1979). Likewise, Langa has a sense of place rooted in its resistance against apartheid and local arts and culture (Booyens et al., 2021). These townships are the best examples of township tourism, which has diversified over the last three decades to include creative experiences, spaces and events (Booyens, 2021; Booyens & Rogerson, 2019a, 2019b). However, creative city strategies do not regard townships as creative neighbourhoods per se, and the participation of township creatives in urban creative economies remains marginal. Formal creative industries are located mainly in central city creative districts in Johannesburg and Cape Town, and small creative clusters are found in certain affluent neighbourhoods (Booyens, 2012; Gregory & Rogerson, 2018; Nkula-Wenz, 2019) (Figures 1 and 2). Minty and Nkula-Wenz (2019, p. 286) contend that the creative economy of Cape Town does not engage with the ‘city’s poorer black and creole peripheries and its unique cultural legacies’. The emphasis of creative city policies has rather been on stimulating creative industries and situating Johannesburg and Cape Town as global cities, as stressed in the literature section.

## 4. METHODS

The findings analysed in this paper draw on my own investigations of township tourism and creativity in addition to participation in related academic, policy and consultancy research. In 2007, I led a study on township tourism in Soweto (Booyens, 2010). I have since observed how township tourism in Soweto, and indeed the township itself, has changed. I have also developed a research interest in creative industries, which expanded to both tourism development and creativity in the townships of Langa and Soweto and participated in an urban policy project for stimulating township economies. In this paper, I build on a suite of eight related papers and book chapters by myself and collaborators (Booyens, 2010, 2012, 2021; Booyens et al., 2021; Booyens & Rogerson, 2015, 2019a, 2019b). This critical mass of previous descriptive research reveals the nature of township creativity. Based on insights from the body of work and field observations over time, it is evident that tourism is closely linked to culture-based creativity in townships. Yet, the path creating role of tourism in township creativity has not been explored to date. In addition to the mentioned published research, data from three projects I was involved in between 2018 and 2022 (Table 1) further inform the analysis presented in the paper. Brief details on the three projects are as follows:

- Project A: a suite of interviews with South African artists conducted with UK-based collaborators. I analysed three interviews with artists either living or working (or both) in townships around Cape Town.
- Project B: my own investigation (interviews, site visits, attending events) on township creativity and tourism, in both Johannesburg and Cape Town. Six engagements were selected for the analysis.
- Project C: creative economies in Cape Town project. I was part of the research team with UK-based collaborators. Two project focus groups, one with creative intermediaries and the other with township creatives in Langa, informed the analysis.

**Table 1.** Research engagements.

No.	Description	City, date	Project
E1	Interview with a beading artist who lives in a township and works in Cape Town	Cape Town, November 2018	A
E2	Interview with a mosaic artist who lives in a township and works in Cape Town	Cape Town, November 2018	A
E3	Interview with a sculptor who lives and works from home in a township	Cape Town, November 2018	A
E4	Interview with a creative practitioner active in townships	Cape Town, April 2019	B
E5	First Thursday networking event for creative township entrepreneurs, hosted in partnership with the provincial government. Thirteen creative entrepreneurs showcased their initiatives and shared their stories	Cape Town, May 2019	B
E6	Focus group with creative intermediaries, including NGOs, arts and cultural organisations/creative practitioners, local government officials and researchers (*N* *=* 18)	Cape Town, June 2019	C
E7	Focus group with creatives, entrepreneurs and facilitators in Langa (*N* = 13)	Cape Town, June 2019	C
E8	Site visit to Guga S’thebe cultural centre in Langa, including an interview with a then City of Cape Town official overseeing cultural sites in townships	Cape Town, June 2019	C
E9	Interview with a researcher from Soweto who did a study on township tourism in Soweto	Johannesburg, October 2022	B
E10	Interview with an academic researcher who has also researched creative industries including Soweto	Johannesburg, October 2022	B
E11	Interview with a researcher who has done township tourism research in Soweto	Johannesburg, October 2022	B
E12	Follow-up interview with respondent E8 who works as a creative intermediary in Cape Town	Cape Town, October 2022	B

Source: Author.

Based on the extant research mentioned and new insights from the projects indicated above, I seek to interpret *why* a rise in contemporary forms of township creativity has occurred in recent years and show *how* tourism and various actors have played a role in the township area change by drawing on evolutionary economic geography theory. When discussing the findings, I consider the risks, vulnerabilities and potentialities associated with township creativity.

I have used my own notes from the mentioned engagements in addition to available interview transcripts to support the analysis presented in this paper. The empirical evidence was analysed thematically in line with the objectives of this research. The interpretation of the evidence is my own original work. The views expressed in this paper are mine and do not necessarily reflect those of any other party. Details of projects, collaborators, institutions and funders follow in end matter section.

## 5. ANALYSIS OF THE FINDINGS

### 5.1. The role of tourism in contemporary township creativity

Strong tourism performance in Johannesburg and Cape Town during the 1990s was followed by the growth of creative sectors and the adoption of creative city policies in the early 2000s (Nkula-Wenz, 2019; Rogerson, 2006). The emergence of contemporary creativity in townships mirrors the abovementioned urban pattern following the dominant service-based economic paths constituting the economic base of these cities (Bhorat et al., 2018; Booyens & Hoogendoorn, 2025; Cheruiyot, 2018). I argue here, first, that the groundswell of township creativity in recent years followed township tourism diversification and, second, that township creativity co-evolved with township tourism. An evolutionary interpretation of these observation-based arguments follows.

**Figure 3.**
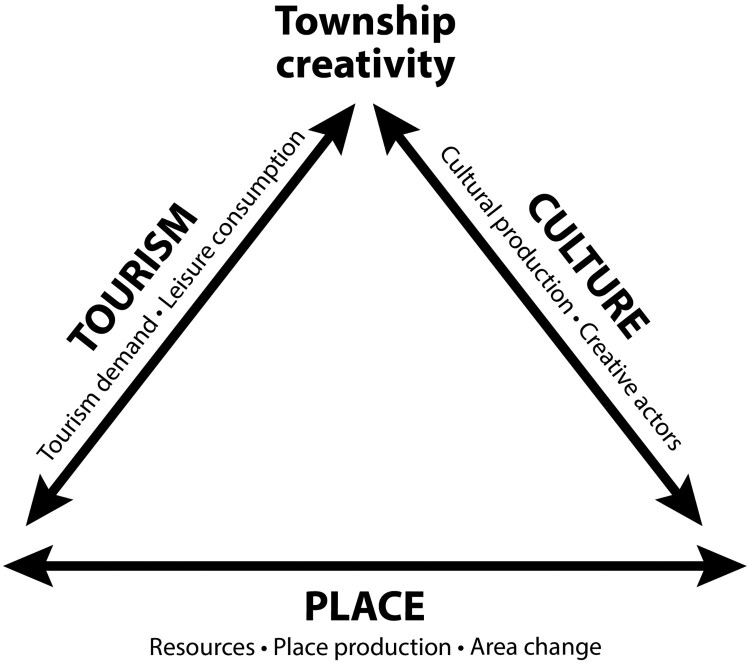
Dimensions of township creativity. Source: Author.

The concepts of relatedness, proximity and path dependence underscore this relationship between culture-based creativity and tourism. The core urban assets of tourism and culture (Malecki, 2010) produce current manifestations of culture-based creativity in townships. These dimensions are place-specific; both township tourism and culture draw on the local resources, that is, the history and heritage (Kourtit & Nijkamp, 2019) and the tangible and intangible culture (E4, E7, E9–E11) of the townships in question. Tourism, accordingly, played a path creating role in stimulating a new, related and complementary activity (Martin, 2009), that is, creativity, driven by tourism demand and leisure-based consumption in townships (Meekes et al., 2017a; Richards, 2011). The emergence of economic novelty occurred owing to the behaviour of individual actors (Boschma & Martin, 2007; Kourtit & Nijkamp, 2019; Martin & Sunley, 2007), that is, township creatives. While tourism and culture rely on place-based resources, these dimensions have a mutually reinforcing relationship with place – contributing in turn to the production of tourism and creative areas, and township area change, as argued in this paper. Figure 3 synthesises the relationship between township creativity and the dimensions of culture, tourism and place.

Key features of township tourism are delineated in Table 2. Creative experiences involve active engagement by visitors in the cultural life of destinations, for example, participation in music and dance or food, art and craft making to co-produce the experiences they consume (Richards & Wilson, 2006). Creative spectacles include music, arts and cultural events. Creative spaces, in turn, are the spatial bundling of cultural activities and are important anchors for creative placemaking (Richards, 2011). In Soweto, a leisure consumption space has developed around the Vilakazi Precinct’s heritage sites, restaurants, shops and venues (Hoogendoorn et al., 2020; Kambule et al., 2024). Elsewhere in Soweto, experience-based tourism; food markets; bars and restaurants; music, dance and theatre performances; and festivals and lifestyle events form part of the broader leisure offering (Booyens & Rogerson, 2019a). Langa boasts vibrant street art and creative experiences (Booyens & Rogerson, 2019a). While functioning as a tourist space, the Guga S’thebe Cultural Centre is also a community arts and culture space (Booyens et al., 2021). Additionally, creative products are made and sold by creative entrepreneurs in both townships.

**Table 2.** Key features of township creativity in Soweto and Langa.

Creative	Soweto	Langa
Experiences	Some participatory experiences (walking and cycling tours; food and beer; street art sites/tours)	Street art tours; creative experiences (art-making; facilitated visits to home galleries of artists; home concerts: African jazz, poetry, storytelling)
Spaces	Creative spaces for shops and events in the Vilakazi heritage tourism precinct; theatre/performance/event spaces	Guga S’thebe cultural centre with creative studios, market stalls and leisure spaces; community performance spaces
Events	Culture and leisure events; music and live entertainment; pop-up food and craft markets; ‘open streets’ initiatives	
Products	Handmade creative goods: bags, jewellery, watches, clothes, caps, t-shirts, shoes, furniture, upcycled products, paintings, printmaking, weaving, beading, mosaics, small sculptures, etc.	

Source: Author.

Township creativity is consumed by foreign and domestic visitors alike, including by a growing Black middle class market (E8–E10). This said, locals and visitors consume township creativity in different ways. According to the available evidence, foreign visitors are particularly interested in creative tourism experiences, while locals and other domestic visitors are more likely to engage in cultural events (Booyens & Rogerson, 2019a). Creative products and leisure spaces are consumed by all visitors and also by locals (E4, E5, E7, E9, E12). For instance, the Vilakazi Precinct becomes ‘party central’ over weekends when younger socially mobile residents from greater Johannesburg flock to Soweto – the place to be seen – to consume ‘Kazi’ culture, that is, contemporary township music and fashion (E9, E10). Furthermore, there is path dependent relationship with the longstanding working-class leisure culture centred on *shebeens* (bars) and dancing venues in townships (Rogerson, 2019; Rogerson & Beavon, 1982). This is illustrative of multiple (complementary) co-evolving paths (Martin, 2009) linked to leisure consumption in townships.

### 5.2. Actors in township creativity

#### 5.2.1. Creative entrepreneurs

From the onset, it should be stressed that creative entrepreneurs are not spatially bound to townships. An active community of creatives are connected to the Guga S’thebe Cultural Centre in Langa (E7), while others are from townships but work in Cape Town (Project A interviews). Township creatives either sell their work in inner-city Cape Town or Johannesburg or at tourism or creative spaces in Soweto and Langa (Projects A and B interviews).

Creative entrepreneurs operate within a neoliberal environment where self-employment is a response to staggeringly high, especially youth unemployment in townships (Charman, 2016; Rogerson, 2019). Entrepreneurship presents opportunities to make a living, as stressed by several interviewees. According to Bongani, a mosaic artist from Khayelitsha (Cape Town township) (E2): ‘People are turning to the art industry because of the unemployment rate. People are tired of looking for a job, they want to create their own things to make their living. It is something that is growing.’

Charman (2016) foregrounds township entrepreneurs who produce music, design flyers, posters, clothes or accessories, and run cafés, restaurants and *shebeens*. Although creative self-employment tends to be survivalist and informal, ‘survivalist micro-enterprises do grow and even become viable businesses’ (Charman, 2016, p. 3). This is consistent with the experiences of creatives in this research, even though they face various challenges and constraints. Most of the entrepreneurs we spoke to wanted to expand their market reach by selling online and elsewhere in South Africa. However, a common discussion point was their continuous struggle to achieve this, owing to the precarity of their self-employment. Township entrepreneurs have volatile income streams, few savings and experience cash flow issues. For instance, Xolile lamented that because of his unstable income, he could not borrow money to sustain and grow his business (E3). Further challenges faced by entrepreneurs include skills to run a business, finding suitable workspaces and accessing buyer markets (E1–E3).

When starting out, creatives typically work from their homes and sell at market stalls – they regard tourists as important buyers of their products (E1, E2, E4, E7). However, creatives maintain that to be competitive, they need the capacity to make goods (quantity) (E5, E7). They, therefore, need spaces to work when they have outgrown their home-based businesses, and the common narrative is that there is a general lack of appropriate workspaces to rent in townships. At the same time, Cape Town’s market stalls and creative workspaces are very expensive and generally inaccessible for creatives from disadvantaged backgrounds with precarious incomes (E8, E12). Yet, young creatives routinely express an aspiration to work and sell ‘in town’ as a measure of success, that is in art galleries, at the V&A Waterfront (a main tourist attraction with high visitor volumes) or the Silo and Old Biscuit Mill (creative) markets (E1–E3, E5, E7). Pop-up markets in townships are not as successful as those in Cape Town, and only selling to tourists in townships is not enough for creatives to sustain their livelihoods (E5, E7). A further challenge creatives face is upscaling from selling at markets to formalising and growing small businesses. Ayanda (E5), a fashion designer, relates how she started sewing at home in a township and then moved to live and work in Cape Town as she became more successful. She then opened a store in central Cape Town, employed five workers and reportedly increased her sales (up to 80%). Another particularly successful fashion designer, Vuyo (E5), shared a similar experience of starting to sew on a township kitchen counter, moving to town, winning competitions, establishing an international fashion brand and exporting to other African countries. Reflecting on his success, he maintained, ‘If you want to take it to the next level, you cannot do it alone.’ Emerging creative entrepreneurs need support to access affordable workspaces, equipment for production, financial investment, business skills and markets, as reiterated by several respondents.

Creative township entrepreneurs engage in both cultural production and creative placemaking. They are makers of a variety of handmade African-inspired arts and crafts. These locally made creative products should be distinguished from imported traditional African ‘curios’ (typically made elsewhere in Africa) also on offer in townships. A creative township practitioner maintains that township creativity is about ‘reinterpreting culture’ through new forms of creativity (E4). Indeed, cultural heritage influences are evident in most, if not all, of the products and practices mentioned. Young fashion designers, active on social media, who focus on street styles (caps, t-shirts, trainers, etc.) draw on ‘Kazi’ culture to brand themselves (E10). Creative makers incorporate cultural expressions in their beading and weaving styles, wax prints used, and images/patterns incorporated in fashion items or artworks. Creative products mentioned, therefore, are hybrid cultural–heritage products that diverge from traditional cultural uses to take on contemporary applications. In addition, creatives maintain that the cultural expressions embedded in their work are also about telling stories (E1, E3, E5). Landiswa (E1) explains that her artwork involves telling stories about rural life in the Eastern Cape (province) and city life in Cape Town. She (and others) adapts traditional beading practices (making necklaces and garments) to work on larger beaded artworks. Creatives are also engaged in participatory experiences involving storytelling, music and theatre performances, facilitating art-making activities with locals, and guides offering street art tours and visits to the home galleries of fine artists. Artists painting murals and making large public mosaics or sculptures are longstanding traditions in both Langa and Soweto. Mural and mosaic artists executing public art are engaged in creative placemaking by physically changing (beautifying) township areas and cultural production alike. The murals and mosaics in Langa convey stories of migrant labour journeys, depict the political struggles of the community and celebrate local heroes.

#### 5.2.2. Local organisations

Creative collectives, local authorities, community arts organisations and creative intermediaries are instrumental in township creativity as discussed here. This research observes that creative entrepreneurs form small collectives^1^ to pool skills and resources to overcome some of the challenges they face. The activities of two such women’s groups are described below. Landiswa (E1) related how their small collective (three ladies) creates artworks collaboratively and run the business together, each taking responsibility for different tasks such as marketing, the finances (bookkeeping), administration and supplies, etc. The other example is the Guga S’thebe Crafters Association, a women’s beading and weaving group (E7). For them, it was not just about selling craft products for income; members who participated in our focus group expressed a strong desire to pass hand skills and traditional practices on to a younger generation. They were involved in schools in Langa to facilitate skills training and community events aimed at youth development (Booyens et al., 2021). Such grassroots community arts organisations, notably, not only have a role in cultural production but also fulfil a social and/or community development function (Booyens et al., 2021; Mbaye & Dinardi, 2019; Sitas, 2020).

Creative collectives and entrepreneurs are backed by creative intermediaries and the City of Cape Town. Development-focused creative intermediaries, often social entrepreneurs engaged in cultural production, are active in townships and the greater Cape Town (E1, E6, E7). They assist creatives with business support, market access and access to appropriate workspaces. For instance, the first collective (mentioned in the paragraph above) is supported by a creative intermediary (arts trust/NGO) brokering international commissions and assisting with the logistics of packing and shipping large artworks. The trust also helped them to register their business and file their taxes. The collective rents studio space from the trust that has a creative coworking space in Cape Town used by several creative groups.

In Langa, several creative makers are tenants of the Guga S’thebe Cultural Centre, funded by the City of Cape Town, which supports several township cultural spaces as part of its arts and culture portfolio (E8). The centre is a multipurpose cultural space with artist studios and market stalls for creative goods. Guga S’thebe’s function as a tourism space, however, is something that certain stakeholders find problematic (E8, E12). The main concern centres on the commercialisation of culture owing to the touristic consumption of creative goods, which reportedly corrodes cultural production and undermines the NGO’s social development (upliftment) work. The creative intermediary focus group participants stressed that the emphasis of cultural policy in South Africa tends to focus on culture as a means to create economic rather than social (public) value (E6). In townships, there is a reported policy confusion about what cultural spaces in townships should be, that is, community or tourism spaces (E8, E12). A disconnect between the activities of the arts and culture, social development, urban planning, and tourism and economic development portfolios at the City of Cape Town is further put forward as a challenge concerning how spaces should be managed and sustained (E8, E12).

## 6. DISCUSSION

Residents are agents of urbanisation in peripheral contexts (Caldeira, 2017; Porreca & Janoschka, 2024). While township creatives stimulate economic novelty from the bottom up, they also contribute to the production of urban spaces as per relational understandings (Comunian, 2011; Kourtit & Nijkamp, 2019; Meekes et al., 2017a). In the context of neoliberalism, urban space becomes a commodity, and informality is influenced by the logic of capital (Avni & Yiftachel, 2014). The privatisation of informality is a risk when informality on public land becomes privatised and marketised, especially when coupled with global property markets (Roy, 2009). Moreover, the touristic commodification of culture is a concern in neoliberal contexts (Richards, 2011; Richards & Wilson, 2006). Recent research points to touristification catalysing gentrification effects such as rising property prices in Soweto’s Vilakazi Precinct, albeit on a relatively small scale, sparked by the entrepreneurial ventures of notably Black Africans (Kambule et al., 2024).

Although this research identifies a close relationship between township tourism and creativity, the risk of creative-led gentrification in townships (Montanini, 2020) is obscure, owing to specific place-based factors. Inner-city creative cluster dynamics, including those underscoring gentrification, differ from what is observed in townships. The rise of creativity in Langa and Soweto is not the outcome of post-industrial urban regeneration, as observed in certain creative districts in Johannesburg and Cape Town where private sector (real estate) investment stimulated gentrification (Booyens, 2012; Gregory, 2016; Nkula-Wenz, 2019). There is currently limited evidence of a private sector push for creative regeneration in townships.

Grodach et al. (2018) argue that the arts-based gentrification narrative is too generalised – how gentrification processes play out is context-specific, and the roles of artists as change agents are not clear-cut. The largely self-directed behaviour of township entrepreneurs in making and selling creative products, along with the bottom-up nature of cultural production in townships – as stressed in this paper – are revealing. Artists/creatives are frequently regarded as agents of gentrification, leading to the displacement of residents (Grodach, 2016; Markusen, 2006). However, artists can both contribute to gentrification and be victims of it, especially in impoverished areas (Markusen, 2006). In the case of the townships, it is unclear at present whether township creativity, including community arts and culture, has led to the displacement of residents, especially by incoming (middle-class) creatives (Scott, 2014). Markusen (2006, p. 1937) maintains that hard to argue that new artistic spaces are displacing anyone, adding that ‘the entry and presence of artists into stable, low-income neighbourhoods does not set off a process of gentrification’. Township creatives are usually existing residents of the township communities. Their relative poverty, along with their need for artistic space, drives ‘sweat equity’ (Markusen, 2006), and many also play an active part in their community, as the findings show.

Young township creatives aspire to work and sell their products in the central city tourism and/or creative spaces. However, they are precarious workers because of their positionality as informal creatives from disadvantaged backgrounds operating on the margins of/out with the social capital governing the formal creative economy. Like other township entrepreneurs, township creatives characteristically operate at a ‘fault line’ between the informal township and mainstream economies while hustling to access various formal and informal resources to generate income (Cooper, 2021). Structural barriers to their participation in mainstream economies remain high (Cooper, 2025). Township creative entrepreneurs are arguably constrained by their *ordinariness*, exemplified by their lack of access to higher education and skills training, financial resources, social networks and institutional connections – all of which matter for successful creative careers (Lingo & Tepper, 2013; Markusen, 2006). The activities of township creatives, therefore, are not necessarily driven by global urbanisation underscored by interconnectedness, competition and technology, and their connections to global flows of creatives and global finances/markets are weak.

Poverty often ‘manifests itself in places where (democratic) social and governmental processes are not well connected to the circuits of neoliberalism’ (Parnell & Robinson, 2012, p. 601), as is the case with townships. Townships, and indeed informal settings, can be regarded as unique liminal spaces for accessing opportunities and social mobility (Cooper, 2021, 2025). Township creatives operate *in between* informal and formal arrangements: institutionally supported/self-directed endeavours, working in creative spaces/street vending, and entrepreneurialism (linked to tourism)/development (linked to cultural policy). Given their developmental imperatives, both public and private role players connected to townships are mindful of the risks of community culture and art becoming commodities to be extracted solely for economic benefits. Contestations vis-à-vis the use of public space for different purposes to the benefit of different groups in townships are evident from the research engagements. Solutions are not straightforward in areas needing economic opportunities to combat poverty in the absence of comprehensive social security nets, typical in Global South contexts.

## 7. CONCLUSIONS

This paper positions townships as the ‘new’ creative spaces in South Africa’s main cities. Seeing creativity in marginalised neighbourhoods extends the spatiality of city creativity beyond innovative clusters dominated by big tech firms (Adler & Florida, 2021) and demonstrates how subaltern creativity is transforming low-income neighbourhoods. The research interrogated the groundswell of township creativity while featuring ordinary creative workers to foreground the situated experiences of informality and neglected realities – of creativity – in Global South cities. Concerning global urbanisation, Follmann et al. (2023) emphasise distinct modes of production in the Global South and call for nuanced understandings of peripheral urbanisation processes as complex – impacted by internal historically and socially situated dynamics and external (global) forces which result in distinct, context-specific urbanisation patterns – as demonstrated in the case of township creativity. In correspondence, Parnell and Robinson (2012) stress the need for nuanced readings of neoliberalism in the urban Global South, privileging the role of social movements (and grassroots organisations) and the agency of local states to foster (community) development. As a Global South example of creative urbanism, this paper demonstrates how individual agents (township creatives) craft their informal livelihoods and how they, along with local organisations (NGOs and local governments), contribute to township neighbourhood change. The contributions of this research to the literature are threefold.

First, this research highlights the path creating role of tourism in catalysing contemporary township creativity and the co-evolving nature of township tourism and creativity. Creativity is driven by tourism and leisure demand/consumption and draws on embedded, place-based cultural and heritage resources (Kourtit & Nijkamp, 2019; Meekes et al., 2017a; Richards, 2011). Contemporary township creativity, therefore, symbolically resonates with local heritage and Indigenous culture, producing material and common spaces with distinctive place identities (Porreca & Janoschka, 2024; see also Roy, 2009), which counters the neoliberal urban visions of creative global urbanism.

The second contribution concerns township creatives creating new forms of urbanity. Bottom-up/subaltern actions are emblematic of peripheral urbanisation (Follmann et al., 2023). While this research acknowledges the precarity and marginality of informal creatives from struggling neighbourhoods concerning their participation in the creative economies of the cities in which they live, they exercise agency by pursuing their interests and translating their ‘hobbies’ into enterprises (Charman, 2016; Cooper, 2021). They are actors who self-organise and stimulate economic novelty while also contributing to cultural production, creative placemaking, their communities in various ways and, ultimately, township area change (Boschma & Martin, 2007; Kourtit & Nijkamp, 2019; Meekes et al., 2017b). Given the associated ambiguities, risks and uncertainties, the case of township entrepreneurs illustrates the complex articulation of agency (Roy, 2009) and underscores the liminality associated with township creativity as a ‘multiple and complex affair’ (Banfield, 2022, p. 615).

Third, this research confirms the vital role of both community arts organisations and cultural intermediaries in supporting community development, creative entrepreneurship and cultural production (Comunian, 2011; Grodach, 2016; Hracs, 2015; Mbaye & Dinardi, 2019). Nuccio and Pedrini (2014, p. 123) stress the importance of cultural production in precarious neighbourhoods to reconstruct social value and enable relational power in communities. This is significant for understanding the symbolic economies of cities and promoting inclusive creative economies. Cities such as Cape Town and Johannesburg, therefore, are encouraged to recognise townships as part of their creative urban economies and as significant spaces of culture and creativity. The latter is typically sidelined in neoliberal contexts overly focused on the economic gains of culture and tourism, highlighted as a concern in townships. Community arts and cultural spaces importantly provide creatives from disadvantaged backgrounds access to workspaces, networks and markets they would not otherwise have (Booyens et al., 2021; Comunian & England, 2022; Markusen, 2006). Such spaces are usually not directly impacted by global markets and privatisation, which arguably ameliorate the risk of creative-led gentrification in townships. The agendas of artists such as township creatives should also not be conflated with neoliberal forces shaping competition in global cities (Markusen, 2006).

Further research is needed on creative urbanism in marginal, low-income neighbourhoods elsewhere in the Global South to shape inclusive creative economies. The roles and agency of ordinary artists/creatives are underexamined. At the same time, the potential exists for new contestations in townships that could erase ordinary lives and create new forms of exclusions. The complexity and contestations associated with creativity in under-resourced communities deserve further attention along with considering appropriate – collaborative and developmental – policy responses to support grassroots creativity, poverty alleviation and neighbourhood improvement.

## Data Availability

Due to the confidentiality of the collected interview and other qualitative data used in this paper, the data are not publicly available.

## NOTE

1. ‘Collective’ is the term used in creative industries for a group of creatives, usually of a similar discipline, working together either formally or informally. This is not a necessarily a form of business such as a cooperative/cooperation.
